# Improved management of farm dams increases vegetation cover, water quality, and macroinvertebrate biodiversity

**DOI:** 10.1002/ece3.8636

**Published:** 2022-03-16

**Authors:** Martin J. Westgate, Clare Crane, David Smith, Colleen O’Malley, Angelina Siegrist, Dan Florance, Eleanor Lang, Mason Crane, Kassel Hingee, Ben C. Scheele, David B. Lindenmayer

**Affiliations:** ^1^ Sustainable Farms Fenner School of Environment & Society The Australian National University Acton Australian Capital Territory Australia; ^2^ NSW Biodiversity Conservation Trust Coolac New South Wales Australia

**Keywords:** artificial waterbodies, agricultural landscapes, management intervention, natural assets on farms, ponds, wetlands

## Abstract

In many farming landscapes, aquatic features, such as wetlands, creeks, and dams, provide water for stock and irrigation, while also acting as habitat for a range of plants and animals. Indeed, some species threatened by land‐use change may otherwise be considerably rarer—or even suffer extinction—in the absence of these habitats. Therefore, a critical issue for the maintenance of biodiversity in agricultural landscapes is the extent to which the management of aquatic systems can promote the integration of agricultural production and biodiversity conservation. We completed a cross‐sectional study in southern New South Wales (southeastern Australia) to quantify the efficacy of two concurrently implemented management practices—partial revegetation and control of livestock grazing—aimed at enhancing the vegetation structure, biodiversity value, and water quality of farm dams. We found that excluding livestock for even short periods resulted in increased vegetation cover. Relative to unenhanced dams (such as those that remained unfenced), those that had been enhanced for several years were characterized by reduced levels of turbidity, nutrients, and fecal contamination. Enhanced dams also supported increased richness and abundance of macroinvertebrates. In contrast, unenhanced control dams tended to have high abundance of a few macroinvertebrate taxa. Notably, differences remained between the macroinvertebrate assemblages of enhanced dams and nearby “natural” waterbodies that we monitored as reference sites. While the biodiversity value of semilotic, natural waterbodies in the region cannot be replicated by artificial lentic systems, we consider the extensive system of farm dams in the region to represent a novel ecosystem that may nonetheless support some native macroinvertebrates. Our results show that management interventions such as fencing and grazing control can improve water quality in farm dams, improve vegetation structure around farm dams, and support greater abundance and diversity of aquatic macroinvertebrates.

## Graphical abstract

Artificial waterbodies such as farm dams are not only valuable for production but also have benefits for biodiversity. We studied the effect of fencing farm dams on vegetation structure, water quality, and macroinvertebrates and found substantial benefits, even in comparative short time periods.

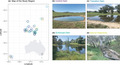

## 1 INTRODUCTION

Numerous ecosystems worldwide are subject to some form of human use, intervention, or management (IPBES, 2019). Although land clearing and land use intensification have already caused species extinctions (Maxwell et al., 2016), a critical step in preventing future biodiversity loss is to identify opportunities where conservation and agricultural production can co‐occur (Leclère et al., 2020). While much research attention has focused on the biodiversity values of uncleared vegetation within fragmented landscapes (Arroyo‐Rodríguez et al., 2020; Haddad et al., 2017; Watson et al., 2018), there are cases where production activities themselves create novel habitats within otherwise intensively managed systems. For example, species that are adapted to early successional states can sometimes benefit from certain forms of timber harvesting (Swanson et al., 2011) or grazing regimes (Moranz et al., 2014). These habitats have the potential to support win–win outcomes where management can support both production and biodiversity.

Freshwater ecosystems are critical areas for biodiversity worldwide, but they are also highly threatened, with numerous rivers having been regulated and wetlands drained and converted to other uses such as agriculture (Reid et al., 2019). Where freshwater elements are retained in modified ecosystems, they often take the form of artificial structures such as farm dams (Malerba et al., 2020). When these artificial waterbodies are managed in a manner that maintains vegetation structure and water quality, they can support substantial biodiversity (Brainwood and Burgin, 2009) (Oertli, 2018). Artificial dams in farming systems can support biodiversity in locations that would otherwise struggle to support diverse biotic communities (Chester & Robson, 2013; Hamilton et al., 2017; Hazell, 2003).

The Murray–Darling Basin, in southeastern Australia, is the nation's most important food‐producing area and supports more than 650,000 farm dams with more than 2.1 GL of water stored, primarily for domestic livestock (Srikanthan et al., 2015). While farms dams can be important for biodiversity conservation (Brainwood and Burgin, 2009) (Hamilton et al., 2017; Hazell et al., 2001, 2004), degraded dams can have significant negative impacts on the environment such as acting as a major source of greenhouse gases (Ollivier et al., 2019). Enhancement of farm dams to improve vegetation cover around and within them could reduce greenhouse gas emissions and improve water quality, in turn enhancing the value of such areas for livestock production (Willms et al., 2002) and biodiversity (Hamilton et al., 2017). However, there is limited information on biodiversity responses to management interventions to improve the condition of farm dams (Lewis‐Phillips et al., 2019). Likewise, there are limited available data comparing the biodiversity of farm dams to that of local natural water bodies (Hazell et al., 2004), so we have no way of knowing the potential of farm dams to support local aquatic organisms.

To address some of these knowledge gaps, we compared the water quality and aquatic macroinvertebrate biodiversity of three categories of farm dams: (1) “control” dams were those where there had been no attempts to improve environmental conditions; (2) Farm dams where a range of environmental works had begun within the past six months, we termed “transition dams”; and (3) dams where grazing control, such as through fencing, had been practiced for at least two years we termed “enhanced dams.” We also added a fourth category to our study, “natural waterbodies.” These consisted of a 200‐m‐long section of creekline with ponded areas and represented the best available “natural” state for waterbodies in our study region. We acknowledge that these ecosystems are intermittently lotic ecosystems and, therefore, differ hydrologically from artificial farm dam ecosystems (Hazell et al., 2004). However, we included them to provide a reference state against which to compare the effectiveness of dam restoration efforts, especially as natural resource managers in our study region have sought to determine how well managed dams perform relative to natural water bodies. Notably, we did not expect farm dams to fully replicate “natural” ponds within creekline systems, although regular dry periods in our study region mean that water flows in creeks and rivers are typically ephemeral (Hazell et al., 2004).

We used the data gathered from the four kinds of water bodies on vegetation structure, water quality, and macroinvertebrate assemblages to address two questions. First, we asked: *Are there differences in vegetation cover*, *water quality*, *and macroinvertebrate assemblage structure between the four categories of water body?* We anticipated that enhanced dams would have higher vegetation cover, lower turbidity and associated impurities, and higher invertebrate richness than control dams due to reduced grazing disturbance; but did not expect them to match natural controls in all attributes. A key part of our analysis was to quantify levels of *Escherichia* *coli* and thermotolerant (fecal) coliforms, as limits for these organisms in water destined for consumption by domestic livestock are set by national guidelines (ANZECC & ARMCANZ, 2000).

While the above approach of comparing different waterbody types is useful, we also were interested to understand which aspects of enhanced dams most strongly influenced variation in macroinvertebrate abundance. This is important because it remains unclear to what extent macroinvertebrates respond directly to increased vegetation cover, versus a combination of increased vegetation cover and improved water quality in farm dams. Therefore, we asked a second question: *What are the inter*‐*relationships between vegetation cover*, *water quality*, *and the abundance of macroinvertebrate taxa?* Answering the two key questions which underpinned this study will provide new insights into the ecological properties of farm dams as a regionally significant landscape feature and how they might be improved by management interventions such as fencing to control access by domestic livestock.

## 2 METHODS

### 2.1 Study area and design

**FIGURE 1:**
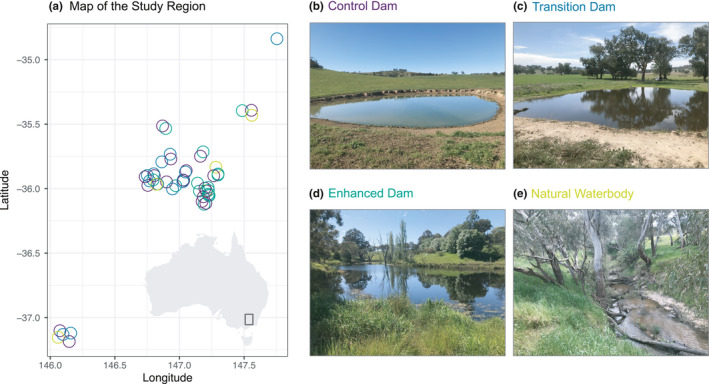
Map of the study region (part a), with study sites shown as hollow circles and colored by type. Points are jittered by up to 0.05 of a degree in both directions to increase visibility of highly proximate waterbodies. Panels b – d show example images of each waterbody type

Our study region encompassed agricultural landscapes of the South West Slopes Bioregion of NSW and northeast Victoria, in southeastern Australia (Figure 1). This area is one of the most modified bioregions in Australia (Benson, 2008). The dominant land use is grazing of livestock for beef, lamb and wool production, and dryland cropping of cereals and oilseed. The predominant breeds of cattle and sheep on our study farms were Black Angus or Simmentals and Merino, respectively.

Our study was a cross‐sectional field‐based empirical investigation (*sensu* Cunningham & Lindenmayer, 2017) which employed a blocked design to contrast water quality and aquatic biodiversity in three categories of farm dams and natural bodies (i.e., connected ponds within a streamline; see Figure 1). Wherever possible, sites within a block were restricted to a single farm to reduce the influence of variability in farm management. Our study design encompassed 62 water bodies across 17 farming properties, in 4 categories.

Our major treatment was *Enhanced dams* (*n* = 21); dams which were either fenced to exclude domestic livestock entirely, or provided a single hardened access point for the entry of livestock from an adjacent paddock. Fences effectively excluded livestock, but did not exclude macropods or lagomorphs. These sites had been replanted or supplemented with native shrubs and trees. There had also been some natural regeneration of reeds and some fringing and aquatic vegetation. Sites selected under this category had been fenced for at least two years. Our requirements for enhanced dam sites were broad as these kinds of interventions are rare in our study area. The extent of revegetation within these fenced areas varied, but typically encompassed the entire fenced area with the exception of the inflow, overflow, and dam wall areas. Dams varied in size, but all were over 1 megaliter in capacity, as smaller dams were considered too ephemeral (i.e., they would likely dry out regularly). To account for the variability among treatment dams, we selected a matched control dam for each treatment dam that had similar characteristics, such as size, shape, position in landscape, and surrounding land use. The area fenced also varied between treatment sites, but were between 50 and 180 meters wide, including the dam.

The remaining dam types were as follows. *Transition dams* (*n* = 12) were those undergoing enhancement and had been totally or partially fenced for no longer than six months at the time of our surveys. *Control dams* (*n* = 24) were those that were unfenced and incorporated into the surrounding paddock. For each enhanced dam and each transition dam, we selected a control dam which had similar characteristics, such as size, shape, position in landscape, and surrounding land use. Control dams were subject to the same management regimes as adjacent paddocks, either livestock grazing, dryland cropping, or both. All dams reliably held water, even in droughts and all dams were designed to capture run‐off (i.e., they were not turkey nest dams or tanks). *Natural waterbodies* (*n* = 5) were naturally occurring and generally permanent water bodies. We specifically targeted connected pond systems within creeklines (lotic systems) for comparison to the lentic farm dam water bodies. For inclusion in the study, natural water bodies needed to be less than two kilometers from the nearest farm dam in a given block. Extensive landscape modification as a result of agricultural development means that such natural waterbodies are rare, hence our small sample size for this category, with not all blocks containing representatives of all four categories of sites.

### 2.2 Field methods

#### 2.2.1 Water

We estimated percentage cover data for vegetation attributes at three zones. We did this by visually estimating the composition of vegetation within the aquatic, riparian, and terrestrial zones of each pond. “Aquatic” vegetation encompassed submerged, floating, and emergent vegetation within the waterbody itself. This was assessed by the observer walking around the dam looking into the water to view any emerging or submerged vegetation within the water, where water clarity permitted. We acknowledge that estimates of aquatic vegetation are constrained by factors such as depth and turbidity, but estimates were made as accurately as possible, and we are confident that these measures provide useful data. We classified vegetation as “riparian” if it occurred between the high‐water mark and actual water level at the time of survey. “Terrestrial” vegetation included all vegetation from zero to ten meters beyond the high‐water mark. We also quantified the woody vegetation at each site within 20 m of the high‐water mark. We did this by estimating the percentage cover of mid‐story and canopy for 0–20 m from the high‐water mark, counting the number of trees that were greater than ten meters in height and the number of trees that were greater than 50cm in diameter and breast height.

We collected three randomly located water samples at two meters from the edge of each water body at a depth of 200 mm and combined all three samples to create one for site analysis. We avoided areas with floating debris and algae. For enhanced dams with hardened livestock access points, we collected water samples adjacent to the access area and around the dam to ensure representative samples of the water in the dam were obtained for analysis. Samples were delivered to a regional water analysis laboratory accredited by the National Association of Testing Authorities (NATA) for processing on the same day as collection. Samples were tested for 11 metrics of water quality using standard analytical methods approved by NATA: electrical conductivity, turbidity, pH, chloride, total nitrogen (consisting of nitrate, nitrite, and Kjeldahl nitrogen), total phosphorus, *E*. *coli*, and thermo‐tolerant coliforms.

#### 2.2.2 Freshwater macroinvertebrates

We sampled macroinvertebrates using a replicated edge sweep method (Gigney et al., 2007) at a subset of 29 waterbodies (14 enhanced dams, 11 control dams, and 4 natural waterbodies) using 300 mm × 300 mm × 500 mm 250 μm sweep nets. Observers completed an initial visual appraisal of a given site to identify each microhabitat that was present within the littoral zone (e.g., emergent or floating vegetation, open water, submerged snags, reeds/rushes). We then sampled these microhabitats proportionally to give a total of 4 × 1 m sweeps at no more than 2 m from the waters’ edge. For example, if the littoral zone comprised 50% open water, 25% snags, and 25% reeds, then we sampled it by doing 1 × 1 m sweep in the area around the submerged snags and another 1 × 1 m sweep around the reeds, and 2 × 1 m sweeps in the open water areas). This process was repeated in three replicates to give a total of 12 sweeps per site per visit. We avoided resampling the same area as previous replicates. The sample we obtained from each replicate was kept separate and sorted individually.

We sorted samples following the agreed level taxonomy (ALT) method (Gooderham et al., 2010). This allowed samples to be sorted in the field and avoided killing and preserving large numbers of specimens. In contrast to most existing methods that restrict identification to a specific taxonomic level (e.g., family), the ALT method classifies each taxon to the most precise taxonomic level that can be reliably identified in the field (Gooderham et al., 2010). We placed the replicate samples into separate shallow 450 mm × 300 mm trays and completed 30‐min timed picks to remove as many different macroinvertebrate taxa as possible (i.e., each dam received a total of 3 × 30‐min sorting periods). This 30‐min period was broken into a 5‐min initial sort, where observers extracted several individuals of the most common species using forceps and pipettes and placed them in an ice cube tray for later identification. The following 20 min was then spent picking out as many taxa as possible. The remaining five minutes was spent focusing on rare or small taxa. We used a results‐based stopping rule whereby if new taxa were detected in the final 5‐min period, we continued for additional 5‐min periods until no new taxa were detected. We checked containers used to store samples for attached annelids, gastropods, and Water Pennies (*Sclerocyphon* spp.) to ensure all organisms were transferred into the final sorting tray. Once the sorting period was complete, we identified taxa according to the ALT Key V1.5 (https://www.waterbugblitz.org.au/cb_pages/files/ALT_KEYS_v1_5_withorderkeyback.pdf) and assigned them to abundance categories. Several representatives from all taxa identified at each site were stored in 70% ethanol solution and were later identified by an experienced observer. This exercise confirmed that the identifications made by field‐based observers were highly accurate.

### 2.3 Statistical methods

#### 2.3.1 Question 1: Are there differences in vegetation cover, water quality, and macroinvertebrate assemblage structure between the four categories of water body?

We used Generalized Linear Mixed Models (GLMMs) to quantify differences in vegetation cover, water quality, and macroinvertebrate assemblages between our four waterbody types. For vegetation cover, we calculated the proportion of area that was covered by any kind of vegetation, then converted this to be unbounded by zero or one via an inverse logit transformation, after setting values that were exactly zero or one to 0.001 or 1–0.001, respectively. We used the log transformation for all of our water quality estimates (except pH), after removal of outliers. We assumed a Gaussian error distribution for all of our vegetation cover and water quality models.

For macroinvertebrates, we modeled richness and total abundance across all macroinvertebrate groups using Poisson distributions with a log link. We then examined the composition of macroinvertebrate assemblages using two separate multivariate regressions. The first regression modeled the presence–absence of macroinvertebrate groups using binomial distributions with a probit link. The second regression modeled abundance of macroinvertebrate groups using negative binomial distributions with a log link. The multivariate regressions used only macroinvertebrate groups found in more than 10 samples (*n* = 14) to avoid overfitting. We included two latent variables to account for residual intergroup correlation.

In all models, we fitted the same set of predictors, namely a fixed effect of waterbody type (a multilevel factor), and a random effect of farm to account for the blocked design of our study. We conducted the multivariate regressions using the Boral R package (Hui, 2016). All other analyses used the lme4 R package (Bates et al., 2014). We visualized the results using ggplot2 (Wickham, 2016), viridis (Garnier, 2018), and ggbeeswarm (Clarke & Sherrill‐Mix, 2017) from the R statistical language (version 4.0.3) (R Core Team, 2020).

#### 2.3.2 Question 2: What are the inter‐relationships between vegetation cover, water quality, and the abundance of macroinvertebrate taxa?

To address our second question, we assumed a causal hierarchy between our different variables, and used this hierarchy to inform a set of models describing the potential associations between them. Specifically, we assumed that vegetation cover could be affected by waterbody type but not by water quality or macroinvertebrates; water quality could be affected by waterbody type and/or vegetation cover; and that macroinvertebrates could be affected by any of the other three parameter sets. We acknowledge that in some cases water quality might affect aquatic vegetation, but not the other two measures of vegetation. We used GLMMs to build a set of competing models for each response variable, and used model selection (Burnham & Anderson, 2002) to choose a best model via the Bayesian Information Criterion (BIC). We maintained the transformations used in our previous stage of analysis, except that our invertebrate models had the abundance of a single invertebrate taxon as the response variable, the identity of the farm dam as a random effect, and a Poisson error structure with a log link.

We compared the model sets using BIC as follows. For vegetation cover, we used our earlier models of vegetation cover as a function of waterbody type and did not employ model selection. For water quality, we selected four response variables that best explained variation in the remaining set, as calculated using the “eleaps” function in the R package “subselect” (Orestes Cerdeira et al., 2020); these were pH, chloride, total nitrogen, and thermotolerant coliforms. We then compared: a null model with no fixed effects; a model that distinguished between dams and natural waterbodies; three models that each contained a single vegetation cover variable (terrestrial, riparian or aquatic); and a model that incorporated the additive or interactive effects of vegetation cover with waterbody type. Finally, for each invertebrate taxon found in more than 10 samples (*n* = 14), we ran 20 models using invertebrate abundance as our response variable. These models were specified as follows: a null model containing only an intercept and no predictors; seven models with only one term per model (waterbody type, vegetation structure in riparian or aquatic zones, or one of our four water quality measures); eight models with additive effects of water quality with vegetation structure; and four models with interactive effects of aquatic vegetation structure with water quality.

To present the results of this analysis, we began by using our first set of GLMMs to calculate predicted mean values of vegetation structure in each of our four waterbody types. We then used these predictions as inputs to our models of water quality variables; and then used those predictions as inputs to our GLMMs of invertebrate abundance. Finally, we compared each predicted value to the corresponding prediction for a control dam, enabling us to state how much a given parameter was higher or lower in the chosen waterbody type than we would expect in a control dam. This approach enabled us to plot a flow diagram of changes in key parameters for each waterbody type.

## 3 RESULTS

### 3.1 Question 1: Are there differences in vegetation cover, water quality, and macroinvertebrate assemblage structure between the four categories of water body?

**FIGURE 2:**
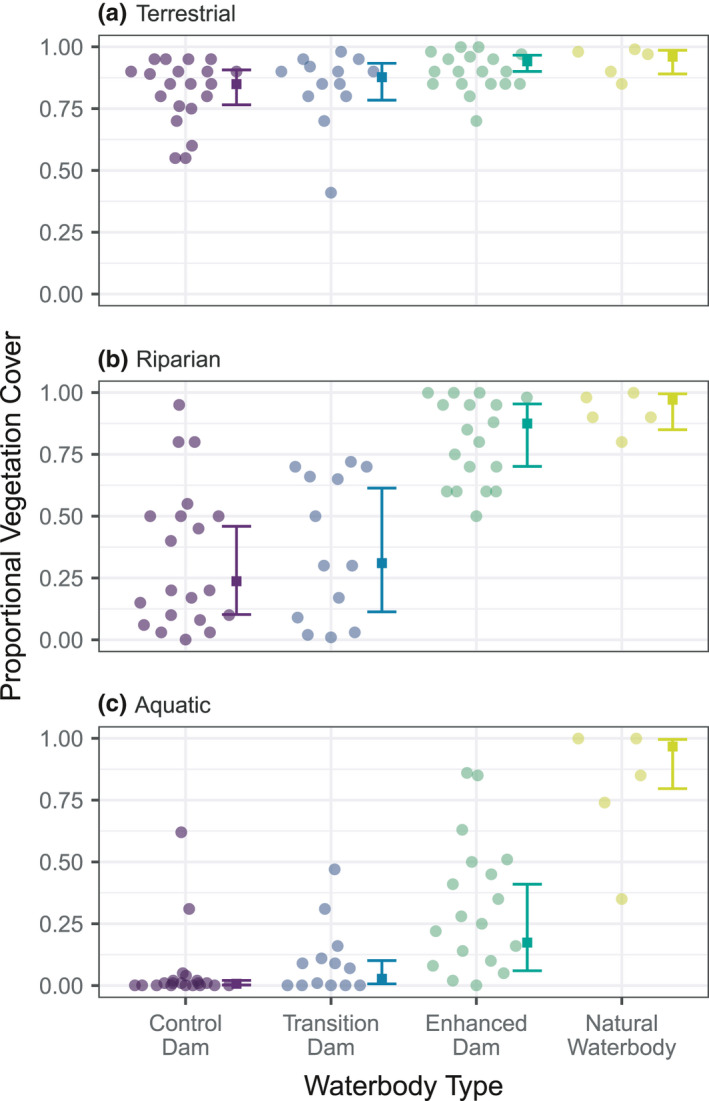
Proportional vegetation cover in three zones (terrestrial, riparian, and aquatic) between our four waterbody types

Vegetation cover associated with our farm dams was typically highest in the terrestrial margin of the dam (mean cover = 86%), and declined in the riparian zone (mean cover = 54%) and aquatic zone (mean cover = 22%). We also found a consistent pattern of lowest vegetation cover surrounding control dams, followed by transition dams, then enhanced dams, with the highest levels of cover found around natural waterbodies (Figure 2). In combination, mixed models show a relatively small difference in mean terrestrial vegetation cover between control dams (85% cover) and natural waterbodies (96% cover), but very large differences in riparian cover (controls: 24%, natural waterbodies: 97%) and aquatic cover (controls: <1%, natural waterbodies: 97%).

**FIGURE 3:**
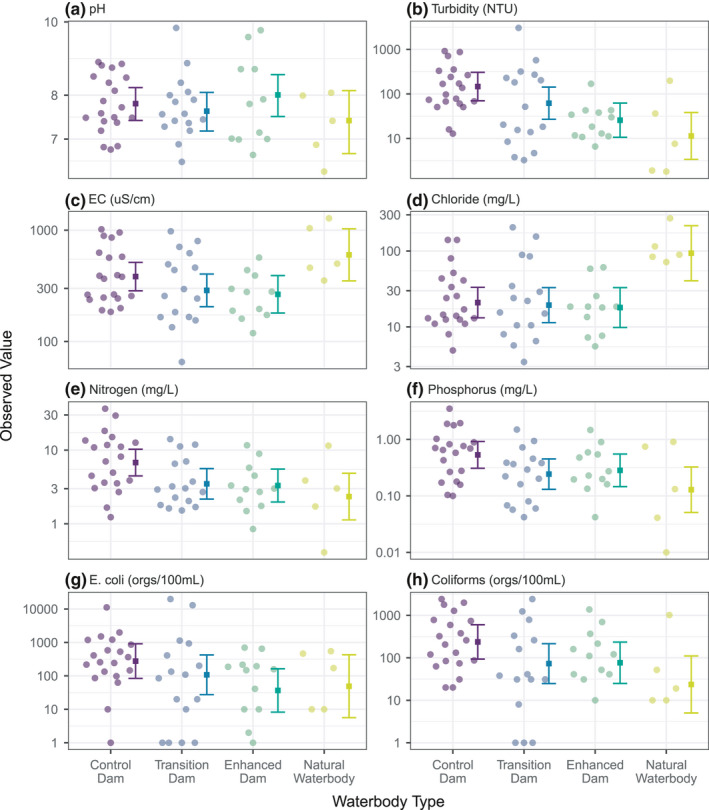
Water quality measures by waterbody type, showing mean and 95% confidence intervals from Linear Mixed Models (LMMs). Note all plots are shown on a log(*y*) scale, but the model for pH was calculated without a log transformation

Our water quality variables were highly correlated (Figure S1), with four variables explaining 87% of the total information in the dataset. These were pH, chloride, total nitrogen, and thermo‐tolerant coliforms. It was unsurprising, therefore, that groups of variables showed similar patterns of variation between waterbody types (Figure 3). Specifically, variables associated with nutrient status (turbidity, nitrogen, and phosphorus) and bacterial status (*E*. *coli*, thermotolerant coliforms) all had their highest values in control dams and their lowest in natural waterbodies. We found that levels of *E*. *coli* and thermotolerant coliforms were extreme in some dams (Figure 3). In Australia, livestock drinking water standards recommend that thermotolerant coliform counts do not exceed 100 organisms/100 mL (ANZECC & ARMCANZ, 2000). This threshold was exceeded by at least an order of magnitude in approximately 65% of control dams in our study. Indeed, there were dams in our study with the highest number of coliforms that it is possible to detect using the test in question (>24,196 per 100 ml), which is over two orders of magnitude higher than the safe threshold.

Our analyses revealed that salinity variables (EC and chloride) did not vary greatly between farm dam categories, but values were significantly higher in natural waterbodies. Finally, pH did not differ in a systematic way between waterbody categories, ranging from neutral to weakly alkaline in the majority of waterbodies.

**FIGURE 4:**
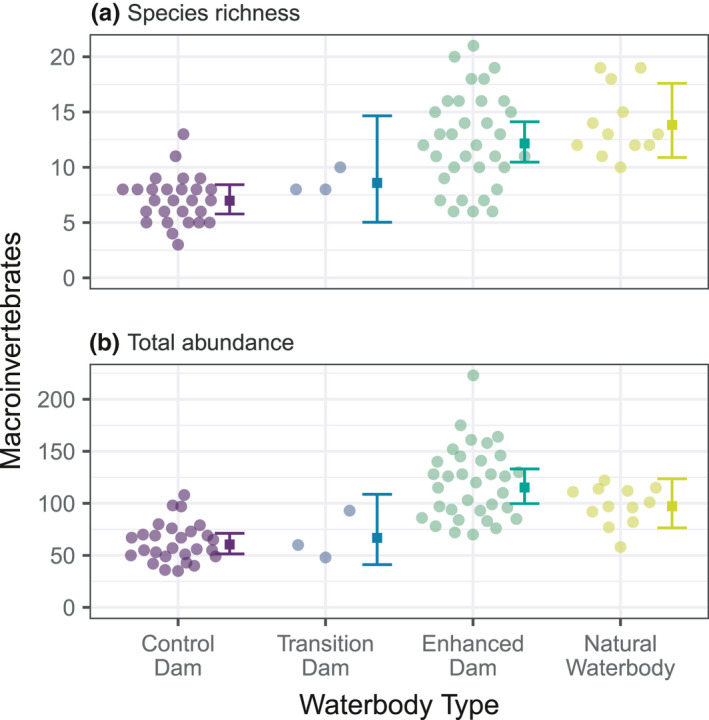
Macroinvertebrate richness (a) and abundance (b) across the four waterbody types

In our macroinvertebrate analyses, we found that enhanced dams supported the greatest abundance and diversity of macroinvertebrates (Figure 4). Observed richness within each sample ranged from 3 to 21 taxa, with the lowest mean richness in control dams (7.0 taxa, 95% CI = 5.8–8.4 taxa) and the highest in natural waterbodies (13.8 taxa, 95% CI = 10.9–14.1 taxa). These results were largely mirrored in our model of total abundance, with the exception that enhanced dams had the highest mean abundance (115.2 individuals, 95% CI = 99.7–133.1), rather than natural waterbodies (97.2 individuals, 95% CI = 76.4–123.6).

Based on our multivariate models of the presence–absence and abundance, we found that enhanced dams supported more suborder Heteroptera, order Trombidiformes, order Odonata, and genus Triplectides than the control dams (Figure S2, left). The enhanced dams also hosted more order Trombidiformes, and the genera Agraptocorixa and Triplectides than the natural water bodies (Figure S2, center). However, the natural waterbodies were characterized by more suborder Heteroptera than the enhanced dams. Our natural waterbodies also supported more suborder Heteroptera, order Odonata, and individuals of the gastropod *Physa acuta* than the control dams. In combination with our earlier findings, these results show that higher vegetation cover and reduced nutrients and bacterial pollutants in farm dams were associated with higher richness and abundance of macroinvertebrate taxa.

### 3.2 Question 2: What are the inter‐relationships between vegetation cover, water quality, and the abundance of macroinvertebrate taxa?

**FIGURE 5:**
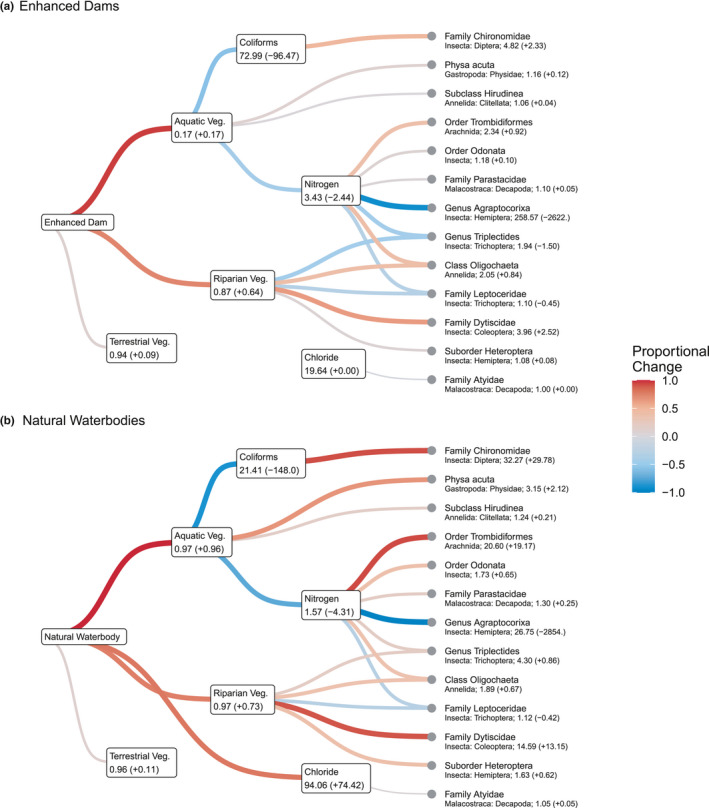
Expected values of vegetation structure, water quality, and macroinvertebrate abundance in enhanced dams (a) and natural waterbodies (b). Lines show positive (red) or negative (blue) effects of variables selected by BIC, while numbers in parentheses show the difference in the expected value of that parameter from the expected value for a control dam. One invertebrate group (Diptera: Ceratopogonidae) has been removed from the diagram for clarity (see text)

Model selection by BIC showed that levels of nitrogen and thermotolerant coliforms were lower in waterbodies with higher cover of aquatic vegetation. Chloride levels were highest in natural waterbodies, and to a lesser extent, in sites with high levels of terrestrial vegetation (although the latter effect was much weaker). Of the 23 macroinvertebrate taxa that were detected sufficiently often to enable statistical modeling, a model which included informative predictors (i.e., not the null model) was selected for 15 taxa (Figure 5). pH was not affected by waterbody type or vegetation structure, and this variable was associated only with the abundance of one macroinvertebrate taxon (Diptera: Ceratopogonidae, or biting midges), and so for clarity we do not display this taxon in Figure 5.

We found that the most influential variable (in terms of number of taxa affected) was total nitrogen (*n* = 7 taxa) followed by percentage cover of riparian vegetation (*n* = 5 taxa). No other variable was associated with the abundance of more than two macroinvertebrate taxa, and terrestrial vegetation was not selected as a predictor for any taxa.

Combining predictions from the final models for all response variables (Figure 5) showed that large increases in riparian vegetation associated with farm dams had a direct effect on macroinvertebrates, increasing occurrence of four taxa and reducing Annelid abundance. The increase in aquatic vegetation in enhanced dams had a greater influence on macroinvertebrates than the increase in riparian vegetation, despite being of lower magnitude. This was because aquatic vegetation was also associated with reduced nutrient levels that were strongly correlated with abundance of a number of macroinvertebrate taxa. Specifically, enhanced dams contained, on average, 45% less nitrogen than control dams, and this difference was itself associated with an increase in abundance of orders Odonata, Trombidiformes, and Decapoda, as well as a 93% decline in Static Boatmen (Genus Agraptocorixa) that were a dominant part of the assemblage in control dams. Increased aquatic vegetation was also associated with decreased coliform levels. This is desirable in itself but also had a positive effect on the abundance of Chiromonidae (nonbiting midges). Increased aquatic vegetation was associated with a small (10%) increase in leech abundance (subclass Hirudinea).

## 4 DISCUSSION

Our empirical study led to three key findings. These were: (1) Management to enhance farm dams, such as by controlling livestock grazing and vegetation disturbance through fencing, resulted in major increases in aquatic vegetation cover as well as changes in a range of variables associated with water quality. (2) Levels of *E*. *coli* and thermotolerant (fecal) coliforms were extreme in some dams, exceeding safe levels (as determined by ANZECC & ARMCANZ, 2000) by over an order of magnitude. (3) Most macroinvertebrate taxa were more abundant in enhanced dams than control dams, but differences in abundance were not related to their indicator value in natural waterbodies (as determined using the ALT measure). This suggests that a modified indicator schema may be needed for farm dams (see also Chessman et al., 2002). In the remainder of this paper, we further discuss these key findings and their significance for farm and wetland management in our study region.

### 4.1 Response to management interventions

Our key finding was that management interventions to control the impact of livestock on farm dams, combined with revegetation and/or conservation/protection of terrestrial plants at some dams, was associated with marked improvements in both vegetation cover and water quality. In addition, we found a strong association between interventions to enhance farm dam condition and high levels of taxonomic richness of macroinvertebrates and a number of individual taxonomic groups. Moreover, we found that combining models of vegetation, water quality, and macroinvertebrate taxa revealed that the greater macroinvertebrate abundance in enhanced dams (Figure 5 ) was associated with a combination of increased vegetation cover and reduced turbidity and nutrient levels (Figure 5). The abundance of several groups in particular, including suborder Heteroptera, order Trombidiformes, and order Odonata, was greater for enhanced dams than control dams. While our study did not investigate the influence of these taxonomic differences on taxa such as frogs, reptiles, or birds, there are examples where increases in the abundance of aquatic invertebrates have been shown to support populations of vertebrate predators (Lewis‐Phillips et al., 2020). Furthermore, improvements in aquatic vegetation similar to those documented here have been shown to have a direct positive effect on a broad range of species and taxonomic groups such as zooplankton (Le Quesne et al., 2020), frogs (Hazell et al., 2001) and fish (http://www.dpi.nsw.gov.au/__data/assets/pdf_file/0019/40663/Fish‐in‐farm‐dams.pdf). Overall, therefore, our results support controlling livestock access to dams through the use of fences to improve the condition of farm dams.

Our macroinvertebrate surveys showed that although farm dam enhancement was associated with an overall increase in the abundance and taxonomic richness of macroinvertebrate assemblages, in practice each taxon responded to different aspects of dam enhancement. We found an association between increased levels of riparian vegetation and lower levels of annelids, for example, which may result from filtering of sediment runoff by riparian vegetation during rainfall events (Sweeney & Newbold, 2014). In addition, our analysis indicated that increased aquatic vegetation was indirectly associated with an increase in numbers of crustaceans via a link to reduced nitrogen (Figure 6). Therefore, while our results support earlier research showing the benefits to freshwater biodiversity of well‐developed riparian and aquatic vegetation (e.g., Fierro et al., 2017; Forio et al., 2020), they also reaffirm the risks of relying uncritically on composite measures of biodiversity such as taxonomic richness or total abundance (Hillebrand et al., 2018).

### 4.2 Water quality in farm dams

A key finding from our study was that levels of fecal coliforms and *E*. *coli* were extremely high in some dams, particularly in unfenced control dams. Guidelines for the quality of water for consumption by domestic livestock in Australia recommend that thermotolerant coliform counts do not exceed 100 organisms/100 ml (ANZECC & ARMCANZ, 2000). This threshold is indicative rather than rigidly enforced, but it was nevertheless exceeded in approximately 65% of control dams in our study. Peak values (>24,196/100 ml sample) were two orders of magnitude higher than this threshold. The effects of such high levels of fecal coliforms and *E*. *coli* on livestock health and on native biodiversity remain unclear. While *E*.*coli* and thermotolerant coliforms are generally not pathogens themselves, their presence is used as an indicator of fecal contamination and hence the possible presence of other waterborne pathogens (ANZECC & ARMCANZ, 2000). Microbial pathogens have been shown to have negative effects on animal performance (Anderson, 1987), but there is evidence that cattle can tolerate high levels of microbial flora (Lardner et al., 2005; Willms et al., 2002). There is only limited published evidence of the effect of poor water quality on animal production, particularly in Australia, where water quantity is generally a much greater concern than water quality. Fecal contamination, however, can affect the palatability of water, which in turn can affect water and feed consumption, optimal rumination, and weight gain (Holechek, 1979; Lardner et al., 2005; Willms et al., 2002).

We found that levels of thermotolerant coliforms and *E*. *coli* were lower on average in enhanced dams than in control dams (Figure 4g). This finding is consistent with recent research from North America, where reducing livestock access to streams through rotational grazing significantly reduced *E*. *coli* levels (Hulvey et al., 2021). Interestingly, transition dams also exhibited a significant reduction of thermotolerant coliforms relative to control dams (Figure 3h), despite having excluded stock for a relatively short period of time (<6 months), suggesting that reductions in pathogens can happen rapidly once stock are excluded.

Beyond our concerning findings regarding water‐borne bacteria, it remains challenging to classify what the environmental and production impacts might be as a consequence of poor water quality in unfenced dams. The majority of water quality indices that we measured either did not have an accepted standard safety limit for livestock that we could find (e.g., phosphorus), or values did not exceed those limits (e.g., salinity). One point not investigated, but that would be worthy of further study, is the risk of biotic effects such as growth of toxic algae that can both reduce palatability of water for livestock (Hyder & Bement, 1968) and potentially impact animal health (Steffensen et al., 1999). An associated environmental risk is that eutrophic dams can release large quantities of greenhouse gases. Indeed, in a recent study, Ollivier et al. (2019) showed that farm dams contribute an order of magnitude more methane per unit area than comparable freshwater lakes and reservoirs. However, CO_2_‐equivalent emissions were dramatically reduced in dams with lower nitrate levels (Ollivier et al., 2019), which is encouraging given our finding that well‐managed dams have lower nutrient levels (Figure 4) than poorly managed unfenced dams, likely due to their higher coverage of aquatic and riparian vegetation (Figure 3).

### 4.3 Natural versus anthropogenic waterbodies

Finally, our work revealed that although enhanced dams displayed many similar properties to natural water bodies, suggesting that management interventions can promote a successful transformation to a better functioning freshwater ecosystem, there also were some large differences. For example, chloride levels and observed/estimated percentage cover of aquatic vegetation were substantially higher in natural water bodies than in enhanced dams. Higher chloride may be a direct result of natural water bodies occurring in lower parts of the landscape, where chloride may accumulate. The higher average percentage cover of aquatic vegetation in natural water bodies may be influenced by differences in geometry relative to enhanced dams (and farm dams in general), with the latter having a much larger surface area and containing areas of much deeper water. Notably, other studies have found inherent differences in several key attributes of vegetation cover (e.g., amount of bare ground, number of trees, height of fringing vegetation, plant species composition) between natural water bodies and artificial water bodies such as farm dams (Hazell et al., 2004; Le Quesne et al., 2020; Reyne et al., 2020). Such differences suggest that natural water bodies may not be an entirely appropriate benchmark for guiding the restoration of farm dams to improve their condition, water quality, and ecological value for biodiversity.

## 5 CONCLUSIONS

Our analyses revealed a consistent pattern of lowest vegetation cover surrounding control dams, followed by transition dams, then enhanced dams (Figure 3). Management to enhance farm dams by controlling livestock access resulted in major changes in aquatic and other vegetation as well as changes in a range of variables associated with water quality. In unmanaged (control) dams, we recorded extreme levels of *E*. *coli* and thermotolerant (fecal) coliforms, that often far exceeded recommended thresholds (as determined by ANZECC & ARMCANZ, 2000). However, management interventions through fencing and control of livestock access led to a significant improvement in water quality with some effects becoming evident within a relatively short period of stock exclusion and revegetation (<6 months).

We found evidence of substantial changes in macroinvertebrate biodiversity resulting from farm dam enhancement. Most macroinvertebrate taxa were more abundant in enhanced dams relative to control dams, and it is possible that management‐generated improved outcomes will flow on to a broader suite of taxa, not measured in this study. Work on other taxonomic groups is required to establish if this is the case. Finally, it is possible that water quality improvements resulting from enhancing farm dams could improve domestic livestock health and productivity (Willms et al., 2002), although experimental trials would be necessary to establish this.

## CONFLICT OF INTEREST

The authors declare no conflict of interest.

## AUTHOR CONTRIBUTION

**Martin J. Westgate:** Conceptualization (equal); Formal analysis (lead); Visualization (lead); Writing – original draft (lead); Writing – review & editing (lead). **Clare Crane:** Conceptualization (equal); Investigation (equal); Writing – review & editing (equal). **David Smith:** Conceptualization (equal); Investigation (equal); Writing – review & editing (equal). **Colleen O’Malley:** Conceptualization (equal); Data curation (equal); Investigation (equal); Project administration (equal); Writing – review & editing (equal). **Angelina Siegrist:** Conceptualization (equal); Investigation (equal); Writing – review & editing (equal). **Dan Florance:** Conceptualization (equal); Writing – review & editing (equal). **Eleanor Lang:** Conceptualization (equal); Writing – review & editing (equal). **Mason Crane:** Conceptualization (lead); Data curation (equal); Investigation (lead); Writing – original draft (equal); Writing – review & editing (equal). **Kassel Hingee:** Formal analysis (equal); Visualization (equal). **Ben C. Scheele:** Formal analysis (equal); Methodology (equal); Writing – original draft (equal); Writing – review & editing (equal). **David B. Lindenmayer:** Conceptualization (lead); Funding acquisition (lead); Supervision (equal); Writing – original draft (equal); Writing – review & editing (equal).

## Supporting information

Fig S1

Fig S2

## Data Availability

Raw data for all analyses in this paper are available via Dryad, doi: https://doi.org/10.5061/dryad.3r2280gj1.
