# Supplementary figures and images for: Improved management of farm dams increases vegetation cover, water quality, and macroinvertebrate biodiversity
Source: Ecol Evol. 2022 Mar 16;12(3):e8636. doi: 10.1002/ece3.8636 (PMC8928867; doi:10.1002/ece3.8636)

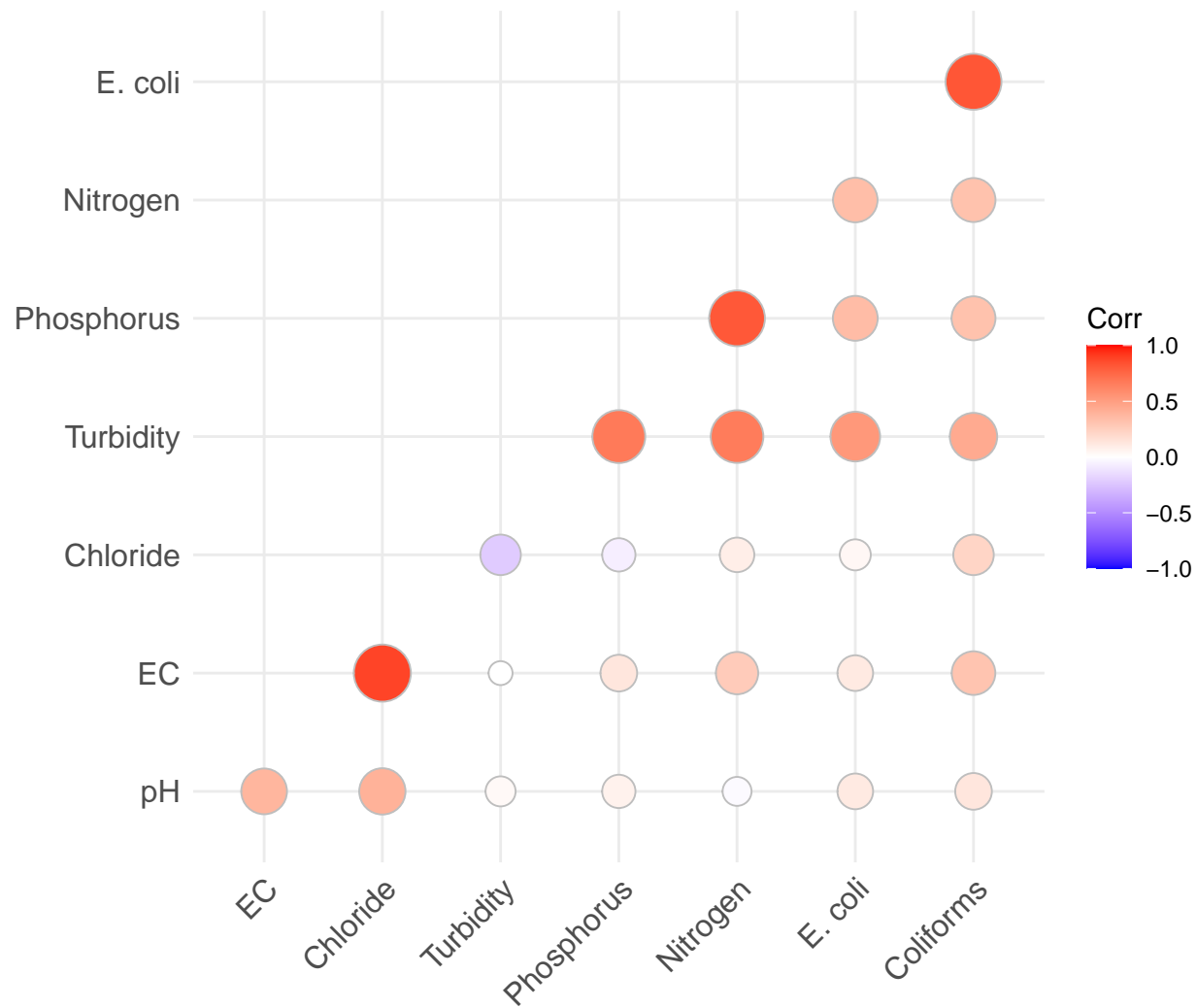

Supplement: Supplementary file 1 — Fig S1 [file ECE3-12-e8636-s001.pdf]

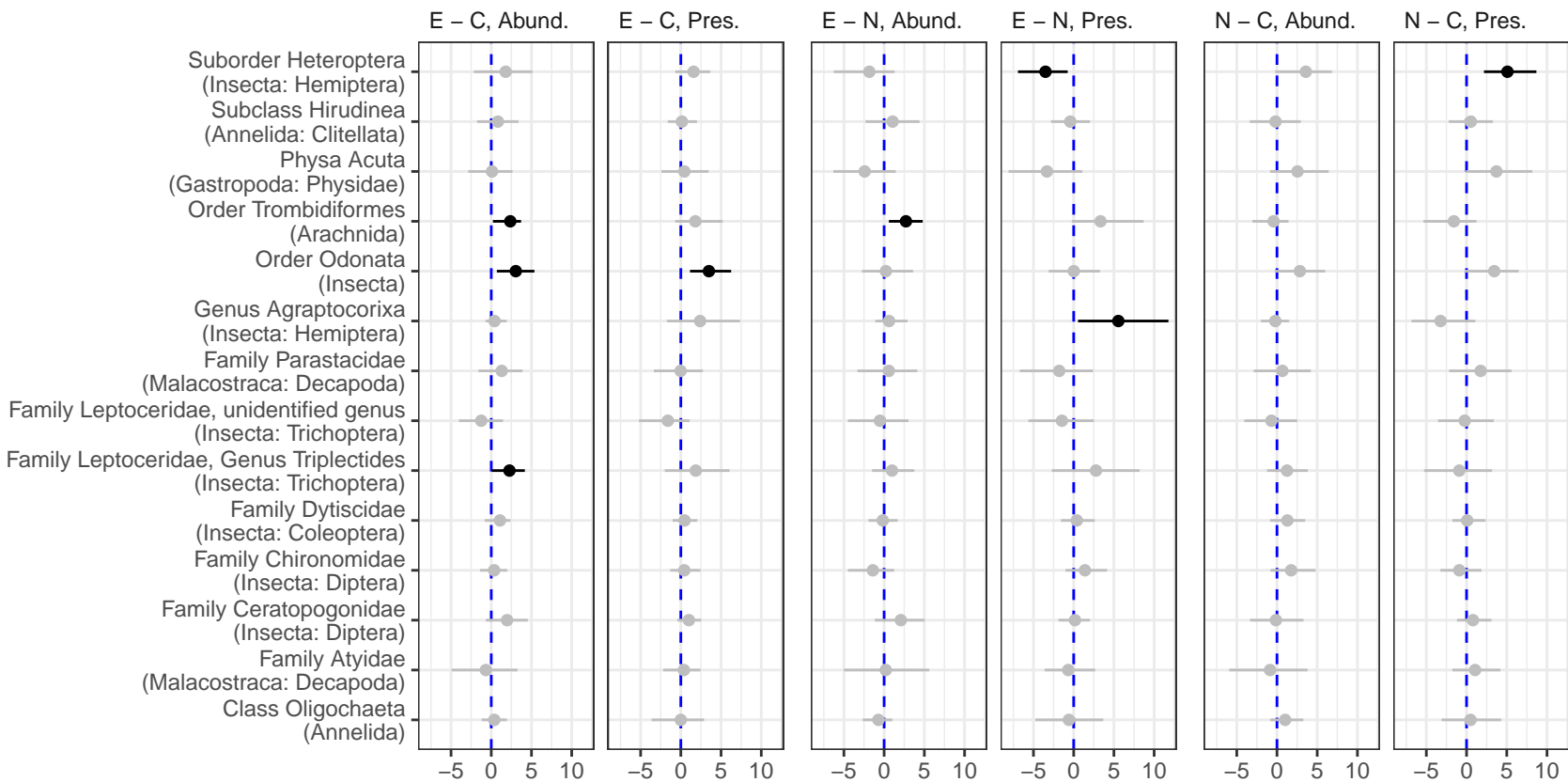

Supplement: Supplementary file 2 — Fig S2 [file ECE3-12-e8636-s002.pdf]
